# Socio-ecological influences on sexual and reproductive health service use: Young adults and provider perspectives from Soshanguve, South Africa

**DOI:** 10.4102/phcfm.v18i1.5272

**Published:** 2026-06-18

**Authors:** Naum M. Maeko, Christo Heunis, Gladys Kigozi-Male

**Affiliations:** 1 Centre for Health Systems Research & Development, Faculty of Humanities, University of the Free State, Bloemfontein, South Africa

**Keywords:** sexual and reproductive health, risky sexual behaviour, socio-ecological model, young adults, service providers

## Abstract

**Background:**

Despite widespread availability of sexual and reproductive health (SRH) services and educational initiatives in South Africa, risky sexual behaviours, including unprotected sex, remain prevalent among young adults. These behaviours drive high rates of unplanned pregnancies, unsafe abortions and sexually transmitted infections. The misalignment between interventions and behavioural outcomes reflects deeper, underexplored systemic barriers – including misinformation, stigma, provider bias and weak policy implementation – embedded within institutional structures and practices.

**Aim:**

Guided by the socio-ecological model, this study explored the factors influencing young adults’ access to SRH services, drawing on the perspectives of both young adults and SRH providers.

**Setting:**

Three public clinics in Soshanguve township, South Africa.

**Methods:**

A qualitative exploratory study design was used. Semi-structured interviews were conducted with a convenience sample of 15 young adults and a purposive sample of five SRH service providers. Data were analysed thematically.

**Results:**

Findings revealed multilevel barriers influencing the use of SRH services by young adults: limited SRH knowledge and misconceptions (individual level); poor parent–child communication and peer pressure (interpersonal level); stigma and religious norms (community level); long waiting times, privacy concerns and provider bias (institutional level) and inadequate youth-friendly policies and restricted access to services like abortion (policy level).

**Conclusion:**

Improving SRH service access and outcomes requires context specific, multilevel interventions aligned to the perspectives of young adults and providers.

**Contribution:**

This study offers a nuanced exploration of SRH service utilisation in an under-resourced setting, generating context-specific insights to inform youth-centred, socio-culturally responsive policy development and service planning.

## Introduction

Sexual and reproductive health (SRH) is recognised as a fundamental human right in South Africa, as outlined in Section 27 of the Constitution, which guarantees access to healthcare, including reproductive services.^1^ To uphold this right, the government has introduced several policies, including the *Choice on Termination of Pregnancy Act No. 92 of 1996*,^2^ the Integrated School Health Policy of 2012^3^ and the National Adolescent Sexual and Reproductive Health, and Rights (NASRH&R) Framework of 2022.^4^ These strategies aim to improve access to contraception, human immunodeficiency virus (HIV) prevention and treatment and safe abortion services, reflecting policy-level commitment to SRH.^4^ However, the translation of these policies into practice, especially in underserved areas like Soshanguve, remains inconsistent.

The NASRH&R framework indicates that SRH services are provided through both the public and private healthcare systems, with the public sector serving approximately 83% of the population.^5^ Public clinics are expected to provide free or subsidised care yet often face institutional-level challenges such as staff shortages, long waiting times and inadequate infrastructure.^5^ Despite the implementation of the Youth-Friendly Sexual and Reproductive Health Services (YFSRHS) model,^6^ many clinics remain ill-equipped to meet young people’s needs due to poor policy implementation and a lack of trained personnel.^6^ These institutional-level limitations impact both young adults seeking care and SRH providers delivering services, shaping the overall experience within clinic settings.

Building on the World Health Organization’s (WHO) Availability, Accessibility, Acceptability and Quality (AAAQ) framework,^7^ insights from Mazur et al.’s^8^ systematic review highlight key evaluative indicators, particularly those relating to accessibility, staff competency, privacy and respectful treatment that are critical for assessing youth-friendly SRH services. Jacobs et al.^9^ provided a grounded application of the AAAQ framework within Zambian tertiary institutions, illustrating how each dimension is experienced in practice. Availability was limited not only by equipment shortages but also by students’ low awareness of existing services. Accessibility was affected by the physical distance of facilities and the inadequate dissemination of information. Acceptability was compromised by concerns about confidentiality, judgemental provider attitudes and the lack of youth-appropriate spaces. Quality was diminished due to insufficient infrastructure and a lack of trained staff.

National SRH data indicate positive trends in contraceptive uptake.^10^ According to the 2016 South African Demographic and Health Survey, about 55% of women aged 15–49 years reported using any contraceptive method.^10,11^ Meanwhile, among sexually active young women aged 15–24 years, current use was approximately 52%, while ever-use was about 68%. However, the disaggregation of data reveals poor utilisation among adolescent girls and young women; in 2019, only 30% of girls aged 15–24 years accessed contraception, and only 44 fifteen-year-old girls accessed abortion services, of which only 20% were obtained in public clinics.^12^ These figures suggest that individual-level barriers, such as low-risk perception, fear, limited knowledge and misinformation, often prevent youth from utilising available SRH services.

To comprehensively examine the multifaceted challenges influencing young adults’ access to SRH, the current study adopted the socio-ecological model (SEM). The SEM was originally developed by Bronfenbrenner in 1979 and adapted for public health by McLeroy et al.^13^ in 1988. The model conceptualises health behaviours as shaped by dynamic and interrelated influences operating across multiple levels: individual, interpersonal, community, institutional and policy.^14,15^

Applying this model, the present study investigated how individual-level factors, including knowledge, awareness and personal beliefs, affect young adults’ engagement with SRH services in Soshanguve. It further examined how interpersonal relationships, particularly the involvement of peers, partners and family, influence young adults’ decisions to seek services. At the community level, the study examined young adults and SRH service providers’ perspectives on how prevailing cultural norms and societal attitudes towards youth sexuality shape young people’s perceptions of SRH and regulate access to SRH services. At the institutional level, the study explored how the structure, capacity and responsiveness of health facilities, including the attitude of healthcare providers, the level of confidentiality and the overall friendliness of services, shape young adults’ experiences and willingness to access SRH services. Lastly, the study assessed how policy-level elements, including legal frameworks, health governance and service implementation strategies, enable or constrain access to quality SRH care. By applying the SEM to examine both user and provider perspectives, this study offers a nuanced understanding of how these interrelated levels converge to shape SRH access and utilisation. In doing so, it provides evidence to inform the design of more contextually responsive, youth-centred interventions in Soshanguve and similarly situated communities.

## Research design and methods

### Design

This study employed a qualitative, exploratory research design to investigate the lived experiences and perceptions of young adults and SRH providers in Soshanguve.

### Setting

Established in 1974, the Soshanguve township, a semi-rural area northwest of the City of Tshwane in Gauteng, reflects rich linguistic and cultural diversity.^16^ Traditionally, the name of the township is said to be derived from the names of the predominant languages spoken, that is, Sotho, Shangaan, Nguni, isiZulu, isiXhosa, isiNdebele, SiSwati and Venda.^17^ More recently, a broader interpretation has been suggested, with Afrikaans and English added to the list.^17^ As of 2024, the population of the township is recorded at 919 748 with most people (75%) being of working age (15–64 years).^18^ Most houses are formal structures, and nearly all have access to basic services like electricity, water and toilets.^19^ However, home ownership is low, with less than half of residents owning or paying off their homes.^16^ About 37% of households are headed by women. Education levels are mixed: very few adults (1.4%) have no schooling, and over 42% have higher education.^17^ Residents trying to access health care in the City of Tshwane face problems such as overcrowded clinics, long waiting times, staff shortages and transport issues.^19^ These contextual factors highlight structural and service delivery constraints affecting SRH access and utilisation, thus making the setting appropriate for exploring barriers to SRH utilisation.

### Study site and population

Soshanguve was chosen for this study because of its large, diverse population and the challenges it faces in healthcare access, especially SRH services. Focusing on three sections, BB, G and JJ, the study aimed to explore how young adults understand and use SRH services and how interventions in the area can be improved. The study took place in three clinics (A, B and C), one in each of the sections. Accurate clinic-level data on the number of young adults attending selected clinics were not publicly available. However, based on the estimated population of young adults in the area and typical clinic catchment sizes, it is likely that these three clinics collectively serve between 20 000 and 40 000 young adults aged 15–24 years in 2023.

### Sample size and sampling

The recruitment sites were purposively selected PHC facilities providing youth-friendly SRH services. All individuals present in the waiting areas who were actively seeking SRH services on the day of the study visit were eligible for recruitment. The following sections describe how participants were recruited and selected in the waiting areas.

#### Young adults

Fifteen young adults within the age group 18–35 years, including seven females and eight males, participated in the study. Five young adults were recruited from Clinic A, six from Clinic B and four from Clinic C. The sampling frame consisted of all eligible young people attending the selected clinic for SRH services during the data collection period. A convenience sampling approach was used to recruit participants from this population. Routine statistics on daily or monthly attendance disaggregated by the study’s eligibility criteria were not available at the time of data collection. Therefore, all eligible attendees present at the clinic during the recruitment visit were invited to participate in the study. Potential participants were approached in the SRH waiting area either before or after their consultation. While participant recruitment occurred at times convenient to participants, inclusion was not based on availability alone but also on eligibility criteria and consent to participate. This recruitment approach ensured that participants had direct and recent experience with SRH services and were therefore well positioned to respond to the research questions.

#### Sexual and reproductive health providers

Five SRH providers participated in the study: one from Clinic A, two from Clinic B and two from Clinic C. Sexual and reproductive health providers were defined as frontline health providers and peer educators directly involved in delivering SRH services to young adults. Purposive sampling was used to recruit participants and ensure the inclusion of participants with relevant expertise and daily experience in delivering SRH services to young people. The providers are specifically trained to provide SRH care and to work with young people. Although small, the sample provided diversity in gender, years of experience and professional background, offering a snapshot of the SRH provider population accessible to young adults in the study area.

### Inclusion and exclusion criteria

All participants were required to be proficient in English, which was deemed appropriate due to its common usage in the region.

#### Young adults

Young adults eligible for inclusion were required to be between 18 years and 35 years of age, to be current users of SRH services at the clinic and to be available during the data collection period. Exclusion criteria included communication impairments, non-South African citizenship and attending the clinic solely to accompany others.

#### Sexual and reproductive health providers

To be included in the study, SRH providers were required to have 6–12 months of experience and be actively working with young people aged 18–35 years within SRH services and to be present at the clinic during the data collection period. Providers working exclusively with other age groups or in SRH departments (antenatal care or labour wards) that were not related to youth-friendly SRH services were excluded. In the study setting, doctors were excluded because they have limited routine involvement in youth-friendly SRH services and are typically only involved when patients are referred. Youth-friendly SRH services are primarily delivered at the first point of contact by nurses, trained peer educators and counsellors.

### Data collection tools

Data were collected between August 2023 and mid-October 2023 using semi-structured interview schedules. The interview guide was developed to explore factors influencing SRH service utilisation. The SEM was used to sensitise questioning and to support the interpretation of findings rather than to predetermine themes. Information sheets and consent forms were also developed. Table 1 provides an overview of interview guides by participant group and SEM level or a priori theme.

**TABLE 1:** Interview questions per a priori theme and participant group.

Theme	Young adults	SRH providers
Individual level: Knowledge and awareness	What do you understand by the term RSB? What are the consequences or potential risks related to RSB? Have you ever heard about SRH services? Which, if any, types of these services have you used? What did you know about SRH services before you used them, and where did you learn this information?	Tell me about common RSB-related issues in your community. Tell me about the key drivers of RSB in your community. How, if in any way, do individual-level factors (personal beliefs, attitudes and experiences) influence young adults’ uptake of SRH services?
Interpersonal level: Family and peers	How do your family members view sexual activity among young adults? How do your peers perceive the use of condoms?	-
Community level: Beliefs and perceptions	How, if in any way, do families or peers influence young people’s access to SRH information and services? How, if in any way, do cultural norms affect young adults’ access to SRH services at clinics?	What are the community’s perceptions of SRH services? How, if in any way, do community or social factors impact young adults’ use of SRH services?
Institutional level: Provision and accessibility	Where do young adults usually seek SRH services in Soshanguve? What barriers, if any, do young adults face when accessing SRH services in clinics? Please share your personal experience with the SRH providers and the clinic you chose. What, if anything, can be done to improve access to and provision of SRH services?	How, if in any way, have SRH services assisted in addressing issues such as sexually transmitted infections (STIs) and unplanned pregnancies among young adults? What has your experience in providing SRH services to young adults been? What method(s), if any, have or has been working for you? What, if anything, could be improved? What messages, if any, are used to communicate about SRH services to inform and encourage young adults to access services? What services, if any, are readily accepted by young adults? Which ones, if any, are not?
Policy level: Rights	Do you believe that all young adults should have access to quality SRH services as a fundamental right?	What is your opinion about South African SRH rights? What policies, if any, work well and what, if any, do not work well.

RSB, risky sexual behaviour; SRH, sexual and reproductive health.

### Recruitment and data collection

#### Access and permissions

Permission to conduct research in clinics was obtained through initial verbal and email communication with clinic managers.

#### Young adults

Participants were recruited from the clinic waiting areas. Interested individuals were escorted to a private interview room designated by facility managers to ensure confidentiality, where they received comprehensive verbal and written information about the study. Upon signing informed consent forms, interviews were conducted in English. A male assistant conducted interviews with male participants to ensure gender-sensitive engagement.

#### Sexual and reproductive health providers

Relevant SRH providers were identified with the assistance of facility managers. After receiving study information and providing written consent, they participated in one-on-one interviews, again in a private room.

#### Data analysis

The data were analysed using thematic analysis, guided by Thompson’s^20^ abductive reasoning framework, which integrates both deductive and inductive logic to capture complex, subjective experiences. The process began with verbatim transcription of all interviews, followed by repeated reading of the transcripts alongside field notes. This step allowed for thorough familiarisation with the content and context of participants’ responses. Coding was conducted manually, drawing both from the SEM as a deductive framework and from subthemes that emerged inductively from the data. Two distinct codebooks were developed, one for young adults and one for SRH providers. Each codebook included definitions, usage instructions and illustrative quotes to ensure consistency and clarity. Related codes were then grouped into themes and subthemes, aligned with the different levels of the SEM. These themes were theorised in relation to existing literature and the SEM framework to interpret emerging behavioural patterns. To deepen the analysis, the analysis explored thematic patterns across young adults and SRH providers, with attention to how perspectives were expressed across gender, without undertaking formal comparative analyses. Findings were organised into thematic tables, supported by illustrative quotes that captured the richness of participants’ experiences. These insights were then woven into the broader discussion, offering nuanced interpretations and drawing out implications for policy and practice.

#### Trustworthiness

Trustworthiness of the qualitative findings was ensured through a comprehensive application of established criteria, including credibility, transferability, dependability and confirmability. To ensure that these methodological principles were rigorously applied, credibility was enhanced through data triangulation by incorporating two sets of perspectives – those of young adults and of SRH service providers.^21^ Transferability was addressed by providing thick, contextual descriptions of the research setting, participant demographics and the socio-cultural landscape of Soshanguve, enabling assessment of the applicability of the findings to other contexts. To ensure dependability, an audit trail was maintained, including comprehensive documentation of the research design, data collection procedures and analytical decisions.^22^ Confirmability was supported through systematic data management and the use of reflective practices, allowing the findings to be clearly traced back to the data and minimising researcher bias. The first author also maintained a reflexive journal to critically engage with her positionality, acknowledge potential biases and enhance transparency throughout the research process.^21^ These strategies collectively reinforced the integrity and rigour of the qualitative inquiry.

### Data management

To maintain data security and anonymity, pseudonyms were assigned, consent forms were securely stored in locked drawers and digital files were password protected and encrypted. These practices aligned with the *Protection of Personal Information Act* (POPIA)^23^ and upheld ethical standards in the handling of research data.

### Ethical considerations

Ethical clearance was obtained from the General/Human Research Ethics Committee (G/HREC) at the University of the Free State (Ethical Clearance number: UFS-HSD2023/0680/4). Written approval was also granted by the Department of Health at both the provincial and district levels. All participants received written and verbal information about the research and provided informed, written consent. Participation was voluntary, and individuals were informed of their right to withdraw at any time without consequences. The researcher had no managerial or supervisory authority over participants, and recruitment was conducted to minimise any potential coercion. Participants’ identities were also protected in transcripts using pseudonyms. Though participants received no direct incentives, they were informed of the potential benefit their input could have on improving SRH services in Soshanguve. Any risk of emotional distress was addressed by ensuring access to a professional psychologist affiliated with the Gauteng Department of Health, should support be required.

## Results

The final sample consisted of 15 young adults aged 22–35 years, with eight males and seven females who had visited the selected clinic sites for services during the data collection period (Table 2), and five SRH providers who were available during this period (Table 3) across the three sampled primary health care (PHC) facilities. The young adults had finished high school and were employed, unemployed or studying (see Table 2). They were recruited in specific clinics (Soshanguve Clinic A, Soshanguve Clinic B and Soshanguve Clinic C) in various parts of Soshanguve, City of Tshwane.

**TABLE 2:** Young adults’ demographic data and pseudonyms.

Code	Pseudonyms	Age (years)	Gender	Educational status	Occupation
**Clinic A**					
IDI M 01	YA9	33	Male	Honours degree	Social worker
IDI F 02	YA7	30	Female	NQF level 3	Unemployed
IDI M 03	YA11	31	Male	Diploma	Programme manager
IDI F 04	YA4	29	Female	Honours degree	Social worker
IDI M 05	YA15	23	Male	Matric (Grade 12)	Student
**Clinic B**					
IDI M 06	YA8	28	Male	Diploma	Public health manager
IDI M 07	YA3	25	Male	Matric (Grade 12)	Welder
IDI F 08	YA5	30	Female	Grade 10	Unemployed
IDI M 09	YA6	22	Male	Matric (Grade 12)	Student
IDI F 10	YA1	24	Female	Higher certificate	Unemployed
**Clinic C**					
IDI F 11	YA2	25	Female	Degree	Teacher
IDI M 12	YA10	32	Male	Matric (Grade 12)	Facilitator
IDI M 13	YA13	35	Male	Matric (Grade 12)	IT technician
IDI F 14	YA12	24	Female	NQF level 4	Student
IDI F 15	YA14	26	Female	Matric (Grade 12)	Student

NFQ, National Qualifications Framework; SRH, sexual and reproductive health.

**TABLE 3:** Sexual and reproductive health providers’ demographic data.

ID	Gender	Age (years)	Level of education	Occupation	Period of providing SRH services
**Clinic A**					
HW 01	F	25	Diploma	Professional nurse	2 years 8 months
**Clinic B**					
HW 02	F	38	Diploma	Professional nurse	Not sure
HW 03	F	57	Degree	Professional nurse	10+ years
**Clinic C**					
HW 04	M	23	Matric	SRH professional	3 years
HW 05	F	23	Diploma	Family planning professional	10 months

SRH, sexual and reproductive health.

### Young adults’ perceptions of the factors influencing sexual and reproductive health service usage

This section presents young adults’ perceptions of the factors influencing their usage of SRH services, organised into *a priori* themes (SEM levels) and subthemes, with illustrative quotes.

### Individual-level factors

Individual-level factors described personal influences on young adults’ utilisation of SRH services, including knowledge, attitudes and misconceptions about SRH services. Five subthemes emerged under this theme, including knowledge of risky sexual behaviour (RSB), adequate knowledge and awareness of SRH, negative attitudes towards SRH services, myths and misconceptions and fear and misinformation related to SRH services.

#### Knowledge of risky sexual behaviour

Participants’ responses reflected a binary pattern of knowledge, where some were informed while others lacked understanding. For example, one participant explained RSB as:

‘Unsafe sex means having sex with somebody not using protection … having sex under the influence of alcohol is also part of unsafe sex.’ (YA1, Age 24, F)

Another participant explained RSB as:

‘According to me the risky sexual behaviour is when a young adult or a young person or any other person is engaging in sexual activities without using protection or when we go out and drinking and then after then we go and engage in sexual activities obvious when you are intoxicated you wouldn’t be using condoms.’ (YA11, Age 31, M)

Some participants demonstrated limited familiarity with the concept of RSB. When asked to describe the term, they were unable to provide a definition until the interviewer clarified its meaning. For example, participant Y14 indicated that she did not know what RSB referred to, prompting the interviewer to explain the concept before continuing with the discussion:

‘Yoh, I don’t have any idea.’ (Y14, Age 26, F)

In response, the interviewer briefly clarified the meaning of RSB before continuing the discussion:

‘So, when we talk about risky sexual behaviour, we’re talking about … it’s sex, but the behaviour we’re talking about is the unsafe way of doing it. So, what are some of the things or actions that you know or examples that you know that involve unsafe sex?’

#### Adequate knowledge and awareness of sexual and reproductive health

However, knowledge about the SRH issue related to RSB was sometimes gained only after negative health experiences. One participant described learning about family planning after experiencing both an STI and a termination of pregnancy:

‘So, I once had STI and also went to ToP, that’s when I learned about family planning. Now I am preventing.’ (YA2, Age 25, F)

#### Negative attitudes towards sexual and reproductive health

Negative attitudes towards sexual health were reported as barriers to preventive behaviour and SRH service utilisation. One participant observed that sexual health issues are often not taken seriously, particularly among older men who perceive themselves as capable of managing their health independently:

‘They don’t take this thing very seriously … taking it very lightly. Some of them don’t use condoms. They usually don’t go to the clinics because they see themselves as old people. Old men who can take care of themselves.’ (YA3, Age 25, M)

#### Myths and misconceptions

Misconceptions about contraceptives were frequently mentioned. Some participants believed that condom use reduces sexual pleasure. One participant said:

‘[*S*]o, there’s a perception that using condoms limits pleasure … Some believe it’s not enjoyable.’ (YA4, Age 29, F)

Fear and misinformation about HIV testing and prevention methods were also reported as barriers to service utilisation. One participant explained that some individuals avoid preventive services because they fear injections and are afraid to test for HIV due to uncertainty about the results:

‘Some of them they are the myth … when they come to do prevention, they will say they are scared of injection. Also, they are scared to check their status because they don’t know … they are afraid because they will not accept it … When they find out that HIV.’ (YA5, Age 30, F)

### Interpersonal-level factors

Interpersonal-level factors described social influences on young adults’ utilisation of SRH services, including family communication, peer influence and fear of social judgement. These interpersonal dynamics shaped young adults’ knowledge, attitudes and decisions regarding the utilisation of SRH services. Three subthemes emerged including the supportive role of families, peer pressure and influence and fear-based perceptions related to social judgement.

#### Supportive role of families

Family settings played an important role in shaping knowledge and access to SRH services. Some participants described supportive family environments where open communication about sexual health helped them understand risks and facilitated access to SRH services:

‘My family was open about discussing these matters and that communication helped me understand the risks and access to [*SRH*] services when needed.’ (YA4, Age 29, F)

Participants indicated that family encouragement positively influenced help-seeking behaviours and access to services:

‘My family was supportive and positively influenced my access to services. They encouraged seeking help when needed, fostering a health-conscious environment.’ (YA4, Age 29, F)

Families influenced knowledge indirectly through media exposure. One participant described how television programmes were used to educate them:

‘[*T*]hey choose television shows such as daily Theta, all of those shows, they talk about health sometimes … They would make sure that we … we watch those TV shows … so that you can get more knowledge and understand this activity better.’ (YA6, Age 22, M)

However, other participants reported limited or shallow communication at home. One participant explained that although sexual health was occasionally mentioned at home, conversations rarely went deeper:

‘In my home, when it comes to speaking about sexual intercourses or sexual diseases … it’s not something that we would sit down and talk about frequently. Yes, we do talk about it, but we don’t go deeper.’ (YA7, Age 30, F)

#### Peer pressure

Peer influence was also described as a controlling factor affecting behaviour. Some participants explained that peer norms shaped attitudes towards condom use and risk-taking:

‘When it comes to peers, it’s like, um, when you’re using condoms, you, you’re afraid, you know, you’re afraid you don’t want to take risks …’ (YA8, Age 28, M)

One participant explained that among some young men, condoms were perceived as reducing sexual pleasure, and peers often encouraged each other to have unprotected sex in order to experience ‘full’ sexual pleasure:

‘The way we perceive it is that you cannot eat sweets being protected. So, our own belief is that in order for the taste of the seeds to fill it, then it means you need to unwrap the cover. So, for us as young men we always believe that a condom prevents us from enjoying the moment the way we’re supposed to enjoy. … that’s why we always encourage each other to say; you need to go meat by meat so that you can feel the pleasure to the fullest.’ (YA9, Age 33, M)

‘Many peers don’t believe in using condoms. They think using condoms reduces the pleasure of sex.’ (YA10, Age 32, M)

#### Fear-based perceptions

Fear of judgement also influenced decisions about accessing SRH services. Participants explained that visiting a clinic for sexual health services could lead to negative assumptions or social labelling within the community. One participant noted that men who visit clinics may be perceived as being sick, which discourages them from seeking care due to fear of community gossip:

‘Normally when a man visits a clinic, they tend to say the person automatically you are sick when you make use of the health services, and in that case, you will be the talk of town.’ (YA11, Age 31, M)

Others reported that seeking STI testing could lead to negative assumptions about an individual’s sexual behaviour. One participant explained that people who go for testing may be judged by others as having engaged in risky sexual activity, such as unprotected sex:

‘Yeah, the attitude is going to be like, did you do something wrong? Why do you feel like you want to go and test for STI? You probably had unprotected sex. That’s why you’d want to.’ (YA7, Age 30, F)

### Community-level factors

Community-level factors described broader social and cultural influences that shape young adults’ utilisation of SRH services, including stigma, cultural beliefs, traditional practices and religious influences. These community norms and belief systems influenced how young adults perceived sexual health, discussed related issues and sought care. Three subthemes emerged under this theme: community judgement and stigma, beliefs in traditional remedies and religious beliefs about premarital sex.

#### Community judgement and stigma

Stigma and fear of judgement were described as barriers to utilising SRH services. Participants reported feeling uncomfortable being seen at clinics or discussing sexual health openly within their communities. A participant noted that community stigma surrounding SRH services discourages open conversations and prevents individuals from seeking care:

‘Unfortunately, there’s still a stigma in our community associated with accessing SRH services. People fear judgement and this hinders open discussions.’ (YA4, Age 29, F)

Similarly, another participant explained that individuals may avoid HIV or STI testing because they fear being judged for engaging in RSB:

‘You know the stigma behind the fact that if you want to go and test now, didn’t play safe? Yeah, I think that’s one of the big things that’s going to happen.’ (YA6, Age 30, F)

Another participant highlighted that cultural norms and stigma related to certain sexual health issues, such as circumcision and STIs, also create barriers to accessing services:

‘Cultural norms, especially around circumcision and STI stigma, make it challenging for young adults to access services.’ (YA10, Age 32, M)

#### Beliefs in traditional remedies

Cultural beliefs and practices were also reported as influencing help-seeking behaviour. Some participants described growing up in communities where traditional remedies were commonly used to address sexual health concerns. One participant said:

‘When we grow up, we were always taught that some of the things can be dealt with by remedies or herbs, among other ways of dealing with [*SRH*].’ (YA9, Age 33, M)

#### Religious beliefs about premarital sex

Religious teachings were also described as influencing attitudes towards sexual behaviour and discussions about SRH. One participant explained that religious teachings emphasising abstinence before marriage shape perceptions of sexual behaviour and are intended to prevent sexual health problems:

‘You know, sex before marriage is a sin. Yeah, I think they’re trying to prevent us from having those kinds of issues.’ (YA11, Age 25, F)

Another participant described how religious beliefs limited open sexual intercourse discussions:

‘I can say you, it’s because of our beliefs, we are Christians, so it’s [*sexual intercourse or problems*] not something that we would sit down and talk about frequently.’ (YA6, Age 30, F)

### Institutional-level factors

Institutional-level factors described influences on young adults’ utilisation of SRH services, including healthcare provider attitudes, privacy and confidentiality, accessibility and operational challenges within healthcare facilities. These institutional conditions influenced how comfortable, supported and willing young adults were to access SRH services. Eight subthemes emerged under this theme: preference for private healthcare services, reactive utilisation of SRH services, men’s preference for traditional health services, positive perceptions of user-friendly services and waiting times, negative attitudes and stigma from SRH providers, lack of privacy and confidentiality, perceived gender bias in service provision, financial constraints and inconvenient clinic hours and long waiting times.

#### Preference for private healthcare services

Some participants expressed a preference for private healthcare services, mainly due to concerns about confidentiality and comfort. One participant explained:

‘I prefer to get these condoms from a private facility because private facilities provide a feeling that everything is confidential.’ (YA10, Age 31, M)

Similarly, another participant noted that this preference was common among young people:

‘Many young people, they prefer private services.’ (YA11, Age 31, M)

These responses suggest that perceptions of privacy and confidentiality strongly influence where young adults seek SRH services.

#### Reactive utilisation of sexual and reproductive health services

Several participants reported that SRH services were often accessed only when problems had already occurred. One participant described using services because there was no alternative:

‘It wasn’t about deciding because I didn’t have a choice.’ (YA2, Age 25, F)

Another participant explained that preventive education and care were often delayed until individuals were already facing health problems:

‘They get this information once they’re in trouble, once already a person is probably pregnant or has been infected with an STI, that’s when now you would get education. Not before, like, we don’t prevent, you know that prevention is better than cure. So, we don’t cure first. They just go there for help already when a person is in trouble.’ (YA6, Age 30, F)

#### Men’s preference for traditional health services

Some participants indicated that men may prefer traditional health services to avoid stigma and judgement associated with clinics:

‘When it comes to STI … men depend mostly on traditional because they believe that in traditional … that’s the safe space where they don’t see … no one is going to stigmatise them …’ (YA10, Age 32, M)

#### Positive perceptions of user-friendly service and waiting times

Although many challenges were reported, some participants described positive experiences with healthcare services, particularly when waiting times were short and staff were approachable:

‘When I visited the facility … the service was user-friendly because the waiting period was not that long.’ (YA11, Age 31, M)

Another participant highlighted differences between facilities:

‘It differs from facility to facility. I made use of services at [*Clinic A*], the service was good. You don’t wait long. But at [*Clinic B*], it differs with days. Yes, there are days where when you go there, I don’t even take time. Then there are days where you even have to carry your own lunchbox.’ (YA11, Age 31, M)

#### Negative attitudes, stigma and judgement by sexual and reproductive health providers

Some participants reported negative experiences with healthcare providers, particularly judgemental attitudes that discouraged service utilisation. One participant described feeling judged by nurses:

‘Sometimes the nurses are not so friendly … A person would just look at you, size you up and say, oh, you’re even this age and now you are feeling that you want to be sexually active …’ (YA12, Age 24, F)

Others also reported unfriendly behaviour:

‘[*T*]he nurses … they’re not friendly. They would like to lash out and not treat you okay.’ (YA6, Age 30, F)

Stigma within clinics was also mentioned as affecting men’s willingness to seek care:

‘Stigma at clinics affects men’s willingness to seek help.’ (YA10, Age 32, M)

Cultural norms were also described as shaping provider and community reactions:

‘You may go to a clinic, and you ask for a condom and maybe you are still a teenager maybe 16 or 15. To other cultural beliefs it’s inappropriate for a teenager to make use of condoms. That means the teenager is already engaging him or herself in sex, which is unacceptable to other cultural beliefs.’ (YA6, Age 22, M)

#### Lack of privacy and confidentiality

Concerns about privacy were frequently reported as barriers to accessing services. One participant explained:

‘When you get to a clinic and then you are all in a hall, that health practitioner will just come and say, those who are here for health related … for sexual health services, just go the side. So, you wouldn’t know if you … want your neighbour to see you go to access those services, that’s why most young people are reluctant to use the service …’ (YA11, Age 31, M)

#### Perceived gender bias in service provision

Some participants believed that SRH services were mainly designed for women, which discouraged male engagement. The participant said:

‘[*S*]ometimes when you talk of sexual reproductive services, you think that it’s one sided, just like … it is meant for ladies, not entirely for men …’ (YA9, Age 33, M)

#### Financial constraints

Financial barriers were also reported as limiting access to SRH services, indicating that economic factors remain an important determinant of access. One participant said:

‘Financial constraints have been a barrier to accessing SRH services … Some individuals struggle to afford even basic health care.’ (YA4, Age 29, F)

#### Inconvenient clinic hours and long waiting times

Operational challenges such as long queues and delays were also described as barriers to accessing SRH services. One participant explained that inefficiencies within clinics, particularly long waiting times, discourage young people from utilising services:

‘Challenges young people face include long queues and delays in service … due to a lack of efficiency in the clinic.’ (YA4, Age 29, F)

### Policy-level factors

Policy-level factors described structural and governance-related influences on young adults’ utilisation of SRH services, including perceptions of access as a right, awareness of policies and recommendations for improving services and education. These policy-related perspectives highlighted opportunities for strengthening health systems and improving access to SRH services for young adults. Six subthemes emerged under this theme: perceptions of access as a right, the need for facility monitoring and evaluation, the need for workshops and training for healthcare providers, the need to facilitate awareness and distribute information, the need to address community stigma through awareness initiatives and the need for school-based SRH programmes.

#### Perceptions of access as a right

Participants believed that access to SRH services should be recognised as a basic right and made widely accessible. One participant said:

‘[*I*]t [*access*] should be given to young people free and very accessible. Young people should actually normalise accessing these services so that we can also deal with the stigma that is attached to young people accessing these services.’ (YA11, Age 33, M)

#### Need for facility monitoring and evaluation

Some participants recommended that healthcare facilities need to implement systems to evaluate service quality. The participants said:

‘They should be given the evaluation form to evaluate the services that they received on that day. And the facility manager should visit those reviews that the clients are making in the remarks.’ (YA11, Age 33, M)

#### Need for workshops for providers

Participants also emphasised the importance of training and capacity-building for healthcare providers. As one participant reported:

‘I think maybe a workshop for nurses or doctors should be implemented by the government.’ (YA11, Age 25, F)

‘I think the community health workers must be capacitated more on the topic itself and also that there should be audits done regularly like this study.’ (YA11, Age 33, M)

#### Need to facilitate awareness and distribute information

Participants identified the need for increased education and awareness campaigns to improve knowledge and access to SRH services. One participant explained that while some young people are aware of certain legal aspects of SRH services, broader knowledge about SRH rights and services remains limited, highlighting the need for more education:

‘Awareness of laws is limited, but there’s knowledge about the legal age for certain services. More education is needed …’ (YA4, Age 29, F)

Similarly, another participant emphasised the importance of community awareness campaigns to ensure that people have access to accurate information about SRH services:

‘Campaign awareness is needed … so that people out there can have more information regarding [*SRH*] …’ (YA9, Age 33, M)

#### Need to address community stigma and awareness

Participants emphasised the importance of addressing stigma at the community level in order to improve access to SRH services. One participant explained that increasing community awareness and education could help reduce stigma and encourage individuals to seek services without fear of judgement:

‘To improve [*SRH service*] access, there should be more community awareness programmes and efforts to reduce the stigma associated with seeking these services. Education is key.’ (YA4, Age 29, F)

#### Need for school-based programmes

Participants also highlighted the importance of introducing SRH education at an early stage through school-based programmes. One participant suggested that providing education in schools, particularly at the secondary level, could help young people gain knowledge before they become sexually active:

‘They should ask for space in schools, then they get to teach them more especially in secondary schools, because that’s where it all starts.’ (YA11, Age 25, F)

### Sexual and reproductive health service providers’ perceptions of the factors influencing young adults’ sexual and reproductive health service usage

This section presents SRH providers’ perceptions of the factors influencing young adults’ usage of SRH services, organised into *a priori* themes/SEM levels and subthemes and illustrative quotes. Service providers described a range of factors that, in their experience, influence how young adults use SRH services. Their perspectives were based on daily interactions with young clients and observations of service utilisation patterns.

### Individual-level factors

Individual-level factors described providers’ perceptions of young adults’ personal knowledge, beliefs, attitudes and behaviours that influence the utilisation of SRH services. These factors were perceived to shape how young adults understand SRH and whether they engage with available services. Three subthemes emerged under this theme: lack of awareness of SRH issues, negative attitudes and behaviours towards SRH education and services and myths and misconceptions about contraceptives.

#### Lack of awareness of sexual and reproductive health issues

Service providers reported that many young adults have limited knowledge of SRH and often seek care only after experiencing sexual health problems such as STIs. One provider explained:

‘Sometimes when they come here, some of them don’t [*know*] anything … most come with STIs, even if they came for family planning … STI is the number one problem in the youth that I work with.’ (HW01)

Another provider observed that some young people believed they already knew enough about SRH and were reluctant to learn additional information, even when education is available:

‘They have this tendency of saying they know whereas they don’t know. And some of these things are caused by them … they don’t want to learn. They don’t want to learn. Because information is there, but they don’t want to learn.’ (HW04)

Similarly, inadequate understanding of prevention methods was also reported, particularly misconceptions about abortion and contraception:

‘They are sexually active, but they don’t have enough knowledge … like to have an abortion is not a prevention method and you need to use a family planning method.’ (HW05)

#### Negative attitudes and behaviours towards sexual and reproductive health education and services

Providers also described negative attitudes among young people towards SRH education and counselling. One provider explained that some young people were described as resisting formal educational approaches and showing little interest in structured health education:

‘[*W*]hat I’ve learned is that we are stubborn … we don’t want to be fed with information. Like number one, what I’ve learned so far is that … [*Distraction*] As I was saying, okay, today’s youth, what they’ve learned is that in order for them to learn you have to force them in a way but in a good way, as I say in an informal way because if you put it in a formal way they will say it is boring and it will be hard for you as a consultant.’ (HW04)

#### Myths and misconceptions about contraceptives

Service providers frequently reported myths and misconceptions about contraceptives as barriers to service utilisation. Myths of weight gain and infertility were commonly mentioned. One provided share that:

‘The myths are the ones that overcome all this … They are going to be fat; they are going to gain weight and all those things. So that’s why they don’t want to use family planning.’ (HW03)

Another provider described fears related to infertility and health complications:

‘They believe when they are using family planning methods, they might not have babies in future, or the blood does not come out so they are going to be sick in future.’ (HW05)

### Interpersonal-level factors

Interpersonal-level factors reflected SRH service providers’ perceptions of how family and peer relationships influence young adults’ utilisation of SRH services. These interpersonal dynamics were perceived to shape young adults’ access to information, attitudes towards sexual health and decisions to utilise SRH services.

#### Poor parental communication

Service providers reported that limited communication between parents and young people contributes to low awareness and delayed utilisation of SRH services. Many young people were described as feeling uncomfortable discussing sexual health issues at home:

‘Most youth are afraid to talk to their parents. You might find that even if I’m a healthcare provider, I’m a mother, but I don’t share information with my child.’ (HW01)

#### Peer pressure and influence

Peer influence was also identified as a strong factor shaping behaviour. Providers explained that young people often adopt behaviours based on peer norms, even when these behaviours increase health risks:

‘They’re doing this because of peer pressure. Somebody is using family plans, and I also should use it even if they don’t have boyfriends, but they still come because somebody is coming.’ (HW01)

Another provider similarly noted that peer group norms influence sexual behaviour:

‘I think it’s peer group pressure. Because most of them, they will tell you that, I was with my friends and what do I do … or I was breaking the virginity, my friends were breaking the virginity and so, it’s just peer group pressure.’ (HW03)

### Community-level factors

Community-level factors described SRH service providers’ perceptions of broader social and cultural influences that shape young adults’ utilisation of SRH services, including stigma, cultural norms and religious beliefs. Three subthemes emerged under this theme: fear of judgement, traditional remedies, and church beliefs.

#### Fear of judgement

Service providers reported that stigma and fear of judgement within communities discourage young adults from seeking SRH services. Young people were described by providers as being concerned about how others might perceive them if they accessed these services. One provider explained that young women who seek family planning services are often judged and assumed to be sexually active:

‘Community judgements are there because when you are a young person and you come for family planning, already they’re thinking you’re busy with boys.’ (HW01)

Another provider described how fear of disappointing parents prevents young people from seeking care:

‘[*T*]hey don’t want to tell their parents; they think their parents are going to be disappointed and so they end up not coming.’ (HW03)

Similarly, service providers reported that fear of being questioned by healthcare staff may discourage young people from visiting clinics for SRH services. One provider explained that some young people worry that they will be interrogated about their reasons for seeking family planning, particularly when they attend the clinic with their parents:

‘Sometimes they fear coming to a clinic, knowing that they are going to be questioned. Why are you taking family planning and coming with your parents, something like that.’ (HW01)

Community perceptions were also described as highly judgemental. One provider explained that young people who attempt to access condoms may be labelled as promiscuous, which discourages them from seeking SRH services openly:

‘Our community takes that so wrong, I wouldn’t say all but some … If they see a young child or adult fetching condoms, they will be like that girl or that boy has started to sleep around in her age … they will say that’s an embarrassment in our community.’ (HW04)

#### Traditional remedies

Service providers also reported that some communities rely on traditional remedies before seeking biomedical care, which may delay treatment:

‘Some families go straight for home remedies before even thinking about the clinic … they don’t always use those services. They only come here when they have serious problems …’ (HW03)

Another provider similarly noted:

‘Some cultures believe in [*traditional*] remedies or herbs.’ (HW04)

#### Church beliefs

Religious teachings were also described as influencing attitudes towards contraception and SRH services. One provider explained that some churches discourage the use of family planning methods, as these practices may conflict with religious teachings or beliefs about fertility:

‘[*T*]here are churches that don’t promote it [*family planning*] … it’s not advisable by the churches … “they believe … they might not have babies in future”.’ (HW05)

### Institutional-level factors

Institutional-level factors reflect SRH service providers’ perceptions of healthcare system influences shaping young adults’ utilisation of SRH services, including service organisation, accessibility, confidentiality and provider practices. These institutional conditions were perceived to influence young adults’ willingness and ability to access SRH services. Four subthemes emerged under this theme: the clinic booking system, clinic operating hours, lack of confidentiality and intentional friendliness and sensitivity by healthcare providers.

#### Booking system

Service providers reported that clinic booking systems sometimes limit access to SRH services. One provider explained that when appointment slots are fully booked, young people may be asked to return another day, which can discourage them from accessing services when they need them:

‘[*W*]e are using the booking system and then they will find that the queue is long and the book is full. We tell her to come next week and then she wants to do it now, now, now, now. And you find that for this week it’s fully booked.’ (HW03)

#### Operating hours

Limited clinic operating hours were also described as a barrier to accessing SRH services. One provider noted that the standard clinic hours may not always align with the schedules of young adults, making it difficult for them to attend:

‘The clinic’s operating hours, from seven AM to four PM, might not always match the schedules of young adults.’ (HW03)

#### Lack of confidentiality

Concerns about confidentiality were also highlighted. One provider explained that clinic procedures may compromise privacy. One provider explained that certain clinic procedures, such as being required to explain the reason for visiting at the entrance, may compromise privacy and discourage young people from seeking services:

‘[*W*]hen they enter the gate, they must tell the person at the gate where they are going so that they can be directed properly.’ (HW03)

Another provider emphasised the importance of maintaining confidentiality during consultations to ensure that young clients feel safe discussing sensitive issues:

‘In a clinic facility or anywhere else, confidentiality is very important … So, meaning what we speak about in this room is in this room. That’s what we need to talk about.’ (HW04)

#### Intentional friendliness and sensitivity

Despite these challenges, providers described efforts to create supportive and non-judgemental environments for young clients. One provider explained that consultations are conducted privately to encourage open discussions and reassure clients that their information will remain confidential:

‘It’s a one-to-one session, so you sit with the patient, and we discuss anything. We assure them that whatever is being said remains here; it doesn’t go outside.’ (HW05)

Another provider noted that using a friendly and respectful approach helps young people feel comfortable asking questions and seeking services:

‘We talk to them nicely, so they don’t feel judged, especially the young ones who are scared to ask questions.’ (HW03)

Another provider noted that using a friendly and respectful approach helps young people feel comfortable asking questions and seeking services:

‘We try to make them comfortable and not judge them because we want them to come back for services.’ (HW04)

### Policy-level factors

Policy-level factors reflect SRH service providers’ perceptions of structural and governance-related influences shaping young adults’ utilisation of SRH services, including access rights, health promotion strategies and community outreach programmes. These policy-related considerations highlight opportunities for strengthening health system responses to improve access to SRH services among young people. Five subthemes emerged under this theme: access to SRH services and rights, health promotion and awareness strategies, the need to improve parent education and awareness, mobile outreach programmes and the role of school health nurses.

#### Access to sexual and reproductive health services and rights

Service providers emphasised that young people have the right to access SRH services without being denied care. One provider explained that healthcare workers are obligated to provide services such as family planning in accordance with patient rights and legal provisions:

‘With our law, I cannot say to anyone no, like patient rights we must give them when they come … asking for family planning, we can’t send them back.’ (HW02)

#### Health promotion and awareness strategies

Providers also described health education activities conducted within healthcare facilities to increase awareness of available SRH services. One provider explained that health promoters regularly provide information to patients in waiting areas about contraceptive methods and where these services can be accessed:

‘We’ve got healthcare providers, health promoters that stand in all the patients waiting in facilities to teach about what methods we have, where to have them.’ (HW02)

#### Improve parent education and awareness

Providers highlighted the importance of educating parents about SRH services in order to support preventive health behaviours among young people. One provider explained that some parents focus on confirming pregnancy rather than supporting preventive measures such as contraception:

‘Many parents, they believe that when you ask them why the child has come, they say “Sister we want to check if she’s pregnant … no we don’t want the child, we’ll do an abortion” … they don’t agree [*with preventive methods*].’ (HW05)

Another provider emphasised that increasing parents’ awareness of SRH services could help them encourage their children to access these services when needed:

‘It [*SRH education*] must be extended to their parents. The parents must be aware of the service and encourage their children.’ (HW03)

#### Mobile outreach

Mobile outreach programmes were identified as effective strategies for improving access to SRH services in communities. One provider explained that outreach campaigns using mobile clinics help reach young people who may be reluctant to visit health facilities:

‘Sometimes we have community campaigns where we go out with mobile clinics for family planning and testing. Those help a lot because some girls don’t come to the clinic on their own.’ (HW02)

Another provider noted that young people may feel more comfortable accessing SRH information and services when they are offered within their communities rather than at clinics:

‘Young people prefer it when services come to them. When they see a mobile clinic in their area, they go for condoms or information because it feels easier and less judgemental.’ (HW04)

Similarly, one provider explained that outreach programmes also include youth-focused activities that create opportunities for open discussions about SRH:

‘During mobile outreaches, we include youth programmes like peer talks and condom demonstrations. It helps them ask questions freely.’ (HW02)

#### Role for school health nurses

Providers also recommended strengthening school-based health services to improve early SRH education and access. One provider suggested that increasing the presence of school health nurses could help deliver SRH education and services directly within schools:

‘I think if we can get school health nurses that start from school and spread them down, down to us. It will be easier.’ (HW05)

### Triangulated summary of the findings

Across all SEM levels, the findings reveal a reinforcing cycle in which individual knowledge gaps are shaped by interpersonal communication patterns, amplified by community stigma and further influenced by institutional practices and gaps in policy implementation. Fear, misinformation and social judgement contribute to delayed and crisis-driven health-seeking behaviours, whereas supportive relationships, respectful service environments and community outreach initiatives facilitate greater engagement with SRH services. The convergence between young adults’ lived experiences and SRH providers’ professional perspectives highlights that barriers to SRH utilisation are not solely behavioural but are embedded within broader socio-cultural and health system contexts. Improving SRH service utilisation therefore requires integrated, multilevel interventions that simultaneously address education, social norms, service delivery practices and policy implementation to promote accessible, acceptable and youth-centred SRH services.

Table 4 presents the triangulated findings, illustrating how factors influencing young adults’ SRH service utilisation operate across SEM levels by integrating perspectives from both young adults and SRH service providers in Soshanguve.

**TABLE 4:** Integrated findings across socio-ecological model levels.

SEM level	Key factors identified	Integrated interpretation
Individual level	Knowledge, attitudes, beliefs, misconceptions	Limited knowledge and persistent myths promote reactive rather than preventive health-seeking behaviour, reinforcing delayed SRH service utilisation
Interpersonal level	Family communication, peer influence, social judgement	Social environments shape beliefs and behaviours; absence of open family dialogue shifts influence to peers, often reinforcing risky norms
Community level	Stigma, cultural norms, religion, traditional practices	Community norms reinforce silence around SRH, increasing stigma and delaying engagement with formal health services
Institutional level	Service accessibility, provider attitudes, privacy, operational barriers	Health system structures either reinforce or reduce barriers; respectful care promotes utilisation, while operational inefficiencies undermine trust
Policy level	Rights, awareness strategies, outreach, education	Policy frameworks exist but require stronger implementation through outreach, education and accountability mechanisms across lower SEM levels

SEM, socio-ecological model; SRH, sexual and reproductive health.

At the individual level, a binary pattern of knowledge, misconceptions and negative attitudes contributes to delayed and reactive health-seeking behaviour. Myths about contraceptives and fear of SRH services discourage preventive practices such as condom use and testing, leading many young adults to seek care only after experiencing unintended pregnancy or STIs. These individual beliefs are shaped by interpersonal influences, including family communication, peer norms and social judgement. While open family discussions can support access to SRH services, limited communication often shifts influence on peers, where risky norms such as discouraging condom use may be reinforced. These interpersonal dynamics are further reinforced at the community level, where stigma, cultural norms and religious beliefs shape attitudes towards sexual health. Fear of judgement and social labelling discourages young people from openly discussing SRH issues and delays help-seeking. At the institutional level, healthcare systems can either reinforce or reduce these barriers. Respectful providers and confidential services encourage utilisation, whereas long waiting times, limited clinic hours, booking systems and privacy concerns discourage young people from accessing care. At the policy level, although policies recognise young people’s rights to access SRH services, gaps remain in awareness and implementation. Participants emphasised the need for stronger outreach programmes, community education, school-based initiatives and improved monitoring of services to address barriers operating across the different SEM levels. Improving SRH service utilisation among young adults therefore requires coordinated interventions that address barriers across multiple SEM levels.

## Discussion

Evidence indicates that access to and utilisation of SRH services among young adults remain limited, a pattern that is also observed in many parts of South Africa.^24,25^ The findings of this study indicate that barriers and facilitators operating across different socio-ecological levels often interact with one another, highlighting the interconnected nature of the influences shaping SRH service utilisation.

Providers in this study reported that young people often sought SRH care mainly during crisis moments, such as suspected pregnancy or STI symptoms, rather than as part of proactive or preventive health behaviour. Many young adults also indicated that young adults tended to delay seeking care due to feelings of fear, embarrassment or limited awareness of available services. This pattern aligns with previous studies conducted in KwaZulu-Natal,^26^ where providers reported similar crisis-driven patterns of service use due to discomfort, stigma and lack of youth-friendly environments.

A major barrier to SRH engagement identified in this study was the persistence of myths and misconceptions surrounding contraception. Providers mentioned that females continue to express fears that contraceptives might lead to infertility or significant side effects such as weight gain. Males shared that condoms were often avoided due to beliefs that they reduce pleasure or undermine masculinity. These findings echo studies from Nigeria^27^ and Uganda,^28^ where contraceptive use was similarly hindered by misinformation and socially reinforced fears. The entrenchment of gender norms and peer reinforcement contributed significantly to the resilience of these beliefs.

Although many participants demonstrated basic awareness of RSB, their understanding of comprehensive SRH services was limited. Participants typically cited informal sources like peers, television or school lessons as primary sources of information. A similar gap in SRH literacy has been noted in studies from Gambia,^29^ where youth relied heavily on non-clinical sources for sexual health information and often misunderstood service availability or functions. This highlights a critical need for earlier, sustained and structured SRH education among young people.

Parental and intergenerational communication about SRH topics was almost absent according to the study participants. Both youth and providers described family discussions on SRH as awkward or taboo, driven by conservative cultural norms. Similar communication gaps were also observed in studies from elsewhere in Ethiopia^30^ and Ghana,^31^ where adolescents relied on peers or media due to limited family support. In many African contexts, authoritarian parenting styles and religious beliefs seem to limit open dialogue and perpetuate secrecy around SRH topics.

Both young people and providers shared that peers emerged as a powerful influence in either reinforcing or deterring SRH service use. Although some positive peer support was noted, the dominant experience was one of ridicule and mockery for those who accessed SRH services or used condoms. For young men, peer networks often valorised risk-taking behaviour and discouraged health-seeking behaviour. These findings resonate with studies from Zambia^32^ and Ghana,^33^ where peer norms shaped masculine identity and stigmatised SRH service use.

Masculinity norms played a particularly strong role in influencing SRH behaviours. Male participants in this study frequently associated condom use with weakness or fear. This aligns with regional and global findings that portray masculine identity as opposed to care-seeking and condom use.^34^ These norms place both male and female partners at increased risk of unintended pregnancies and STIs and require targeted intervention strategies.

Among both young adults and providers, stigma was a recurring theme across participant narratives. Merely being seen at an SRH facility was perceived as shameful or indicative of risky behaviour. Youth feared being labelled promiscuous or HIV-positive by peers or community members. These concerns mirror findings from studies in KwaZulu-Natal^35^ and Ghana,^33^ where stigma reduced SRH service uptake and created additional psychological burdens for adolescents seeking care.

From the views of both young adults and providers, cultural and religious beliefs further shaped how young people viewed SRH services. Some participants preferred traditional remedies or spiritual interventions, believing these to be more trustworthy or less shameful than biomedical care. These findings align with research in Nigeria^15^ and rural South Africa^36^ that documented widespread reliance on traditional or spiritual practices as alternatives to modern contraception and clinic-based care. However, evidence also highlights important gender differences: in Nigeria, women’s contraceptive choices were often constrained by partner opposition and unequal decision-making power within households.^5^ In South Africa, adolescent girls emphasised the need for confidential, respectful contraceptive services, and boys more often highlighted stigma and the importance of HIV testing and counselling.^36^

Religious teachings were described as a major barrier, especially among unmarried youth. Premarital sex was often considered sinful, and seeking contraceptives was equated with immoral behaviour. Some providers acknowledged that religious values discouraged both conversation and access. Similar patterns have been found in Nigeria^15,37^ and South Africa,^38^ where religious conservatism undermined youth access to SRH services. Nigerian girls were disproportionately restricted by cultural and religious expectations around premarital sex, while boys’ access was shaped more by peer-related stigma and perceptions of promiscuity. In South Africa, health-seeking behaviours among adolescents in Soweto also showed that girls were more likely to avoid services for fear of community and religious disapproval, while young men cited stigma but demonstrated greater mobility in seeking HIV-related care.^15^

Several participants, particularly young men, reported feeling excluded or overlooked by health systems that appeared to cater more to girls and women. Health providers confirmed that male youth were underrepresented in service design and delivery, and their unique needs often went unaddressed. This echoes findings from South Africa^38^ and Nigeria,^39^ where adolescent boys felt unwelcome in SRH spaces and perceived these services as irrelevant to their needs.

Both young adults and providers shared that confidentiality breaches and fear of being seen by other community members accessing services further deterred young people from visiting clinics. Concerns about judgement from neighbours and providers, particularly in facilities without private entrances or youth-friendly environments, were widespread. Previous research from South Africa^35^ and Kenya^40^ has emphasised that lack of anonymity and poor clinic design contribute significantly to underuse of services by youth.^35,41^ In Kenya, young females who sought contraception risked being labelled ‘spoiled’, facing moral judgement and reputational damage, while boys experienced less scrutiny but were still influenced by peer norms around masculinity and sexual behaviour.^40,42^

This study noted that young people reported operational barriers, particularly long waiting times, which discouraged timely and consistent use of services.^43^ In South Africa’s Eastern Cape province, one study reported that clinics are often overcrowded, with long queues.^43^ As a result, individuals were frequently advised to return on another day, which discourages timely and consistent access to services. Similar findings were observed in studies from other parts of sub-Saharan Africa, where long waiting times were common and often led to discomfort and discouragement among individuals seeking SRH services.^44^

Providers further pointed out rigid appointment systems and clinic hours that clashed with school schedules, which further frustrated adolescents and limited consistent access. These findings are consistent with study conducted in Cape Town, where adolescents default on contraception because appointment times are inconvenient and clash with school hours.^45^ Clinics operate early morning only; after-school visits are often turned away. Long waiting times reduce utilisation.^46^ One study in Limpopo province evidenced that rigid scheduling and long distances to clinics worsened this impact. For instance, services had dedicated hours or slots in the afternoon for youth-friendly services (14:00–17:00), but individuals from distant villages would arrive late and often miss those slots.^46^

Provider attitudes varied considerably. While some providers were described as empathetic and supportive, many participants, particularly girls, felt judged or moralised for seeking SRH care. Similar concerns have been noted in other sub-Saharan African studies, where providers’ judgement was identified as a major barrier to adolescent engagement with SRH services.^6,14^ However, contrasting findings from the Western Cape suggest that where providers offer non-judgemental, youth-friendly care, SRH service use improves significantly.^47^

Considering policy-level factors, both young adults and providers emphasised that access to SRH services should be viewed as a fundamental right rather than a privilege. Participants proposed several solutions to improve access and reduce stigma, including the use of mobile clinics, community outreach and better integration of SRH education into schools. Mobile SRH services were perceived as effective for reaching remote or reluctant youth and also in addressing stigma and confidentiality fears. These findings are supported by evidence from studies conducted in South Africa,^48^ Zambia^49^ and Zimbabwe.^50^ In South Africa, it was found that mobile clinics (the Tutu Teen Truck) were more acceptable among young people (16–24 years).^48^ The mobile service attracted younger clients, more men and had higher HIV diagnosis rates than nearby fixed clinics.^48^ In Zambia, community-based and peer-led interventions (including mobile or outreach type services/hubs) significantly increased uptake of SRH services among adolescents and young people.^49^ In intervention zones, service use was much higher than in control zones.^49^ In Zimbabwe, mobile outreach expanded access to long-acting and permanent contraception, which improved the quality of counselling and increased choice for clients through outreach that took services closer to users.^50^ In all these studies, users rated the mobile clinic very highly compared to conventional clinics.

Collectively, these findings underscore the need for youth-centred SRH services that address intersecting individual, interpersonal, community, institutional and policy barriers. Policies and interventions must not only expand access but also transform the environments, social, familial and clinical, in which young people make decisions about their SRH and uptake of services for such.

This study has several limitations that warrant consideration. Firstly, the research was conducted within a relatively small number of health facilities and involved primarily nurses, peer educators and counsellors. This narrow sample may constrain the transferability of the findings to other settings or to broader interprofessional contexts. Secondly, the qualitative design prioritised the exploration of participants’ perspectives rather than facilitating direct comparative analyses such as differences by gender or between young adults and providers. Additionally, reliance on self–reported data introduces the possibility of social desirability bias.

Future research would benefit from engaging a wider range of SRH providers, including doctors and programme managers, across more diverse settings. Incorporating comparative approaches may further enhance the evidence base needed to inform effective SRH programme design and implementation.

### Recommendations

Based on the SEM, this study recommends a multilevel approach to address the SRH needs of young adults in Soshanguve. At the individual level, interventions should be culturally informed and tailored through analyses that capture the lived realities of youth, ensuring support that is both personalised and accessible. At the interpersonal level, fostering open dialogue within families is essential, and safe spaces for communication must be created to normalise discussions around SRH. At the community level, awareness campaigns should be critically evaluated and strengthened, with peer networks mobilised to promote healthy sexual behaviours and counter negative norms. At the institutional level, cultural, gender and economic barriers must be directly addressed by funding inclusive and youth-friendly services within clinics, ensuring confidentiality and non-judgemental care. Finally, at the policy level, sustained attention is required to reduce financial and stigma-related barriers, alongside the establishment of regular evaluation mechanisms that ensure services remain responsive to young people’s needs. Collectively, these recommendations underscore the importance of a holistic and integrated strategy that operates across all five SEM levels to improve SRH access and outcomes in the local context.

## Conclusion

This study demonstrates that improving SRH service utilisation among young adults in Soshanguve requires more than just service availability. It demands systemic transformation across social, institutional and policy conditions. The findings show that individual knowledge gaps and risk perceptions are reinforced by family silence, peer norms, community stigma and clinic-level practices that undermine confidentiality and trust. Together, these intersecting barriers create structural conditions that discourage preventive care and sustain reactive, crisis-driven health-seeking behaviour among young adults. The convergence of young adults and provider views reflects that service challenges are not solely behavioural but are embedded within service delivery systems and implementation processes. Thus, there is a need for youth-centred models of care that should prioritise dignity, privacy, male-inclusive programming and provider capacity-building alongside strengthened school- and community-based outreach. This study used a socio-ecological approach to demonstrate the need for integrated SRH interventions that address multiple, interconnected barriers to service access and use. Such an approach is critical in health systems for advancing equitable, responsive and sustainable SRH services in township and similarly resource-constrained settings.
